# Influence of Nano-Hydroxyapatite on the Metal Bioavailability, Plant Metal Accumulation and Root Exudates of Ryegrass for Phytoremediation in Lead-Polluted Soil

**DOI:** 10.3390/ijerph14050532

**Published:** 2017-05-16

**Authors:** Ling Ding, Jianbing Li, Wei Liu, Qingqing Zuo, Shu-xuan Liang

**Affiliations:** 1 College of Chemistry and Environmental Science, Hebei University, Key Laboratory of Analytical Science and Technology of Hebei Province, Baoding 071002, China; 18730271511@163.com (L.D.); auhlw80@126.com (W.L.); sunqc710@163.com (Q.Z.); 2 Environmental Engineering Program, University of Northern British Columbia, Prince George, BC V2N4Z9, Canada; Jianbing.Li@unbc.ca

**Keywords:** nano-hydroxyapatite, ryegrass, Pb-polluted soil

## Abstract

Lead is recognized as one of the most widespread toxic metal contaminants and pervasive environmental health concerns in the environment. In this paper, the effects of nano-hydroxyapatite (NHAP) on remediation in artificially Pb-contaminated soils and ryegrass were studied in a pot experiment. The addition of NHAP decreased the water- and acid-soluble, exchangeable, and reducible fractions of Pb, extracted using the Community Bureau of Reference (BCR) method, whilst greatly increasing the residual fraction of Pb. Oxidizable Pb was increased slightly. No significant increase in soil pH was caused by the application of NHAP. Compared to conditions without NHAP, the addition of NHAP decreased the Pb content in ryegrass shoots and roots by 13.19–20.3% and 2.86–21.1%, respectively. Therefore, the application of NHAP reduced the mobility and bioavailability of Pb in the soil. In addition, the application of NHAP improved the fresh weight of shoots and roots, and promoted the growth of ryegrass. NHAP played a positive role in stimulating ryegrass to secrete tartaric acid.

## 1. Introduction

Soil pollution by heavy metals has become a serious concern in many developing countries due to intense industrialization and urbanization. Heavy metals are more complex than other environmental pollutants because they can be toxic to all living organisms. They are not biodegradable and tend to accumulate in tissues [1]. Lead is recognized as one of the most widespread toxic metal contaminants and pervasive health concerns in the environment [2]. It is generated from the natural weathering of rocks and industrial activities, including mining and lead ore smelting, lead-acid battery manufacturing, lead-based paints, etc. [3]. In addition, Pb is not an essential nutrient in the metabolic processes of plants and animals, and it can accumulate to high levels and have biological toxicity to organisms [4,5]. The limit for Pb content is 35 mg/kg, according to the environmental quality standards for soils [6]. Therefore, the development of remediation strategies for Pb-contaminated soils is very important for human health and ecological protection.

Currently, most researchers focus on the use of chemical remediation and phytoremediation to control soil heavy metal pollution [7,8]. There have been a number of studies on in situ immobilization of Pb-contaminated soils using hydroxyapatite, and two different mechanisms were mainly found: Dissolution–precipitation and ion exchange (between Pb^2+^ in solution and Ca^2+^ on hydroxyapatite lattice) [9]. The influence of both is dependent on pH and pore solution chemistry [10]. In general, hydroxyapatite has a better effect in acidic soil (pH ~ 5), but the soil in our experiment was alkaline soil (pH ~ 8). Therefore, hydroxyapatite did not apply in our experiment. Nanomaterials have a higher reactivity and adsorption capacity than ordinary-sized materials. Nano-hydroxyapatite (NHAP) is an important mineral component of human hard tissues, such as bones and teeth, and is the less soluble form of phosphate. It is an ideal material for the immobilization of heavy metals because of its high sorption capacity for heavy metals, low water solubility, high stability under reducing and oxidizing conditions, availability, and cost effectiveness [11]. At present, the application of nano-hydroxyapatite on Pb-contaminated soils is limited. The purpose of this study was to evaluate the effectiveness of nano-hydroxyapatite in immobilizing Pb in contaminated soils. Compared with most hyperaccumulators, ryegrass is preferentially used for phytoremediation because it is extensively grown, easy to be managed, and has a high biomass, therefore, it is economical to use it for phytoremediation [12]. Ryegrass can accumulate a large amount of toxic substances, and has a high tolerance to heavy metals [13]. Thus, for this experiment we selected ryegrass as a phytoremediation plant.

Our previous work studied the effect of 0.5% (*w*/*w*) NHAP on Pb-polluted soil, and the results showed that NHAP reduced the Pb contents of ryegrass [14]. However, at present, there are few studies on the immobilization of Pb using NHAP; especially, there is no research about the combination of NHAP and ryegrass, and the reasons for the reduced mobility and bioavailability of Pb caused by NHAP have not been fully investigated. In this paper, we studied the effects of higher NHAP applications at different Pb contents in the remediation of Pb-contaminated soil. The aim of this study was to investigate the effects of NHAP on the changes in the form of Pb in soil, and the accumulation of Pb in ryegrass and the growth of ryegrass.

## 2. Materials and Methods

### 2.1. Design of the Pot Experiment

The tested soil (0–20 cm) was farm field soil extracted from Baoding City, Hebei Province, China. The soil was air-dried, crumbled and then milled (2 mm). The soil had a pH of 7.87, 23.28 g/kg organic matter, 8.43 g/kg total nitrogen (TN), 7.62 g/kg total phosphorus (TP), and a cation exchange capacity (CEC) of 1.40 mol/kg. The pH was measured on a 2:5 (*w*/*v*) water suspension of the soil sample after stirring for 10 min [15]. TN in the soil was determined using the Kjeldahl method [16]. The organic matter in the soil was determined using the potassium dichromate volumetric method. Total phosphorus was determined using the molybdate–ascorbic acid procedure at 700 nm [17]. Cation exchange capacity was determined using the compulsive exchange method with 1 mol/L of ammonium acetate (pH 7) [18]. The background value of Pb in the soil was 58.28 mg/kg. Pb was applied to the soil as Pb(NO_3_)_2_ at four concentrations (0, 400, 800, and 1200 mg/kg of dried soil). Lead-spiked soil was aged in a greenhouse for one month. Nano-hydroxyapatite was purchased from the Nanjing Emperor Nano Material Company (Nanjing, China) and had a purity greater than 96%. The pH of NHAP was 8.11, and the specific surface area was 154 m^2^/g. The pH was measured on a 1:20 (*w*/*v*) water suspension of the nano-hydroxyapatite samples after stirring for 1 h [19]. The Brunauer–Emmett–Teller (BET) surface area of the NHAP was determined by N_2_ sorption analysis at 77 K in a surface analyzer after degassing.

The pot experiments were conducted in a greenhouse with an air temperature of 22–25 °C at Hebei University. The design of the pot experiments for the different treatments is listed in Table 1. Twenty seeds of ryegrass were sown per pot, which was filled with 0.15 kg soil (60% moisture content) and 1.5 g NHAP. Pots without NHAP were used as a control. Three replicates were set for each treatment. Thirty days after germination, the samples of ryegrass were harvested by cutting the shoots at the soil surface, and the roots were carefully separated from the soil. Plants were thoroughly washed with running water, followed by distilled water, and then dried at 105 °C for 1 h, and then at 65 °C in an oven (BGZ-30, Shanghai Boxun Industry and Commerce Company, Shanghai, China) until completely dry. They were finally weighed, and the dry weight of the plants was recorded [20]. The soil was air-dried, crumbled and then milled (2 mm).

### 2.2. Pb Content Determination

Shoot or root dry matter (0.1 g) was digested using 5 mL HNO_3_ and 2 mL H_2_O_2_. Soil samples (0.1 g) were digested with 5 mL HNO_3_, 2 mL H_2_O_2_ and 2 mL HF. The Pb concentration in the digested solutions was determined using an A3 atomic absorption spectrophotometer (Beijing Purkinje General Instrument Co., Ltd., Beijing, China). The standard reference material (GBW 07411, National Institute of Metrology, Beijing, China) was analyzed with the samples during the course of the analyses. The linear correlation coefficient of the Pb standard solution was *r* > 0.999. The mean recovery of the Pb standard reference material was 98%. The range of the Pb standard solution was 1–15 mg/L.

### 2.3. Determination of pH

Four grams of soil sample were put into plastic centrifuge tubes. Then, 10 mL of distilled water was added to the tubes. The mixture of soil and solution was stirred for 10 min and then allowed to settle for 30 min. The pH value was measured using a pH meter (HACH, Loveland, CO, USA).

### 2.4. Organic Acids Analysis

A portion of the ryegrass rhizosphere soil was collected during the collection of the plant samples and kept at 4 °C for analysis. For organic acid extraction, 1.0 g of soil was extracted by 10 min of agitation at 200 rpm with 10 mL 0.1 mol/L H_3_PO_4_, and the extracts were filtered using a 0.2 mm filter membrane. The separation of organic acids was carried out on a system consisting of an analytical high-performance liquid chromatography (HPLC) unit (Waters 1525 Binary HPLC Pump, Waters 2998 Photodiode Array Detector, Waters, Milford, MA, USA) with a Cosmosil packed column (C18-PAQ, 4.6 mm I. D., Nacalai Tesque, Kyoto, Japan), in conjunction with a column heating device set at 30 °C. Elution was carried out isocratically at a solvent flow rate of 1.0 mL/min of 0.02 mol/L NaH_2_PO_4_ and chromatographic-grade acetonitrile (98:2). The injection volume was 20 μL. Detection was performed with a UV detector set at 213 nm. The standard solution was prepared by mixing eight low molecular weight organic acids: tartaric acid, lactic acid, acetic acid, citric acid, pyruvic acid, oxalic acid, succinic acid, and l-malic acid. Organic acid identification was performed by comparison of the retention times with those of authentic standards. The peaks in the chromatograms were integrated using a default baseline construction technique. The organic acid was quantified by the peak area.

### 2.5. Community Bureau of Reference Sequential Extraction Tests

The Community Bureau of Reference (BCR) was used to extract different fractions of Pb [21]. One gram of dried specimen of soil sample was added to a polypropylene centrifuge tube; the sequential extraction procedures are listed in Table 2.

### 2.6. Statistical Analysis

All values are the means of three replicates. Data are presented as the mean value ± standard deviation. To verify the statistical significance of differences among the treatments, data were analyzed using SPSS statistical software (IBM Nederland BV, Amsterdam, The Netherlands) using one-way ANOVA and Duncan’s multiple-range test. Differences were considered significant at *p* < 0.05.

## 3. Results and Discussion

### 3.1. Speciation Analysis of Pb in Soil

The use of sequential extraction furnished detailed information regarding the origin, mode of occurrence, biological and physicochemical availability, mobilization, and transport of heavy metals [22]. In this paper, BCR was used to evaluate the effect of NHAP on the changes of Pb fractions in the soil. It has been reported that conventional hydroxyapatite can react with Pb to form chloropyromorphite during the sequential extraction process (especially in the non-steady amended state) [23]. However, at present, the sequential extraction method is still a universally-applied method for the determination of heavy metal fractions. We attempted to avoid this possible error in the experiments by strictly controlling the experimental conditions, and placing the results of the BCR measurements in uniform and comparable conditions. According to the degree of heavy metal bioavailablity in different metal fractions, metal species were divided into three categories: bioavailable, potentially bioavailable, and bio-unavailable. The bioavailable category included the water soluble and exchangeable fractions. The content of this heavy metal portion was small, but had excellent mobility, and was most likely to be absorbed and utilized by organisms [24]. The mobility of heavy metals was directly related to water solubility, and the high water solubility of heavy metals can result in a high leaching risk for groundwater and can threaten the health of organisms [25].

**Figure 1:**
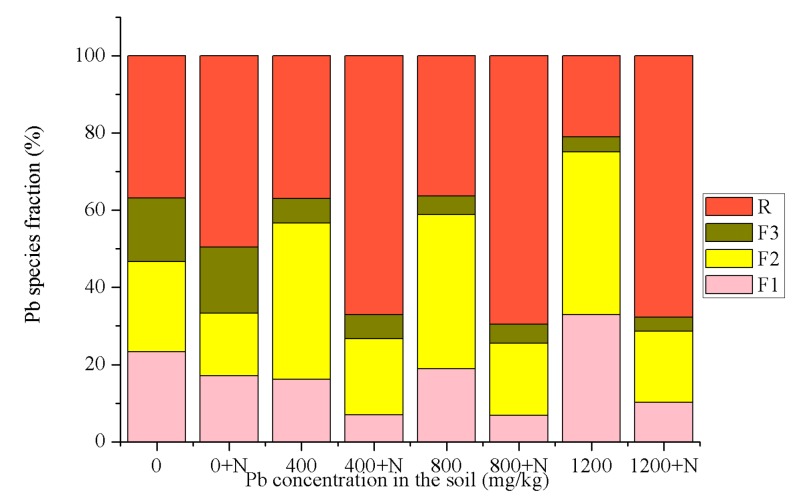
Lead partitioning in Pb-spiked soil with and without NHAP application. The values of 0, 400, 800, 1200 respectively stand for the addition of Pb content (0, 400, 800, 1200 mg/kg); 0 + N, 400 + N, 800 + N, 1200 + N respectively stand for the addition of Pb content (0, 400, 800, 1200 mg/kg) and NHAP (1.5 g). The operationally defined soil fractions were: (F1) exchangeable, (F2) reducible (iron/manganese oxyhydroxides), (F3) oxidizable (organic matter and sulfides), and (R) residual. N: nano-hydroxyapatite.

As shown in Figure 1, the amounts of Pb present in F1 and F2 were noticeably lower after NHAP was added compared with the control group, declining by 21.69–66.08% and 25–52.02%, respectively. The residual fraction of Pb increased by 124.67% compared with the control group. After adding NHAP, the Pb content of F3 increased by 6.83% on average. Any changes for F3 were not as obvious as those of the other three Pb fractions. The results showed that the application of NHAP can change the fraction of Pb from bioavailable to bio-unavailable. NHAP significantly reduced the mobility and availability of Pb in soil. The portions of the four Pb fractions extracted using BCR were almost the same as soil with different applied Pb levels. The formation of pyromorphite from Pb was the most important effect of NHAP application [26]. This led the transformation of Pb from non-residual to residual fractions by changing its dissolution–precipitation mechanism. NHAP was first dissolved in soil solution which released phosphate ions, and then phosphate ions and lead ions in the soil solution produced a low solubility type of lead phosphate. Nanoparticles have a very large micro-interface, with a strong surface complexation ability with respect to heavy metals, which accelerates the rate of dissolution, shortening the equilibrium time between dissolution and sedimentation. The acidity required by dissolved nanoparticles was lower than larger particles, which can reduce the probability of acidification as secondary pollution in the processes of hydroxyapatite to immobile Pb. Therefore, nano-scale materials are expected to improve the remediation effect. It was reported that the bioavailability of heavy metals in soil are linearly related to their biotoxicities [27]. The results showed that the application of NHAP could alleviate the biotoxicity of Pb and lower its mobility in soil to ensure the healthy growth and development of plants.

### 3.2. Effect of Nano-Hydroxyapatite on the pH of Soil

The pH of soil is an important parameter that affects metal immobilization and dissolution in soil. Metal solubility and mobility decreased with the increase in pH. According to Table 3, the addition of NHAP increased soil pH by 0.02–0.13 units, compared with the control group. However, this difference was not significant. The result was consistent with the finding that the application of NHAP can increase the soil pH value [3]. NHAP was dissolved in the soil solution, releasing PO_4_^3−^, and PO_4_^3−^ to react with the H^+^ in the soil-generated HPO_4_^2−^ and H_2_PO_4_^−^ [28]. Soil pH decreased with the rise of Pb content in soil, but there were almost no significant differences. For the 1200 mg/kg treatment, the pH of the soil had a larger reduction. In addition, the roots of plants can also affect soil pH by secreting protons and organic acids. The contents of heavy metals in soil have impacts on the secretion of plant roots [29]. Therefore, the observed tendency in this study might be the result of factors such as plant secretions, NHAP, and Pb content. More in-depth research to determine the specific impact of each factor on the pH of soil is needed.

### 3.3. Effect of Nano-Hydroxyapatite on Pb Accumulation in Ryegrass

**Figure 2:**
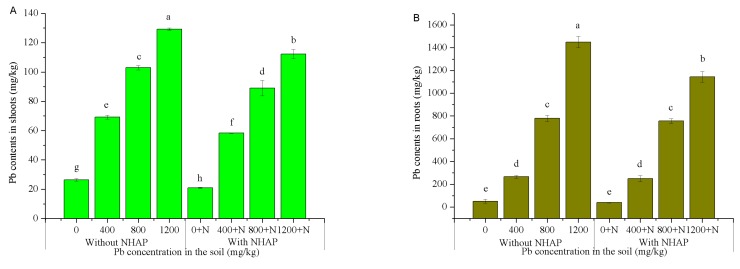
Effects of NHAP on Pb contents in shoots (**A**) and roots (**B**). a–h: The different letters in the figure represent significant differences between treatments at *p* < 0.05.

The Pb content in shoots and roots are shown in Figure 2. The results showed that the metal content in shoots and roots was altered by the addition of Pb to the soil, as well as the addition of NHAP. Increasing concentrations of Pb in soil led to an increase of Pb content in roots and shoots. Compared to the control group, the addition of NHAP led to an approximately 2.86–21.1% decrease in Pb concentrations in the roots, and a 13.19–20.3% decrease in the shoots. There was a significant decrease in shoots with NHAP treatments compared to the control group, while the decrease caused by NHAP was not significant in roots, except at the highest concentration of Pb contamination. Plant-available Pb was highly correlated with water-soluble Pb (*r* = 0.812 for shoots, *p* < 0.05; *r* = 0.870 for roots; *p* < 0.01). Thus, the application of NHAP decreased the Pb concentration of roots and shoots, because NHAP converted the bioavailable fractions of lead into bio-unavailable fractions.

### 3.4. Plant Growth and Biomass

**Figure 3:**
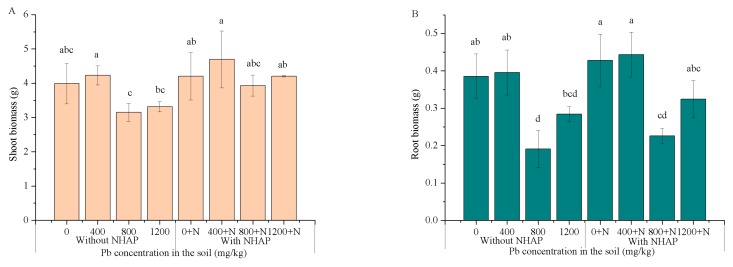
Effect of NHAP in different treatments on ryegrass shoot (**A**) and root (**B**) biomass. a, b, c, d: The different letters in the table represent significant differences between treatments at *p* < 0.05.

In this study, ryegrass grew rapidly and healthily, with no visual symptoms of necrosis or whitish-brown chlorosis during plant growth. After the plants were harvested, the height of ryegrass and the fresh weight were measured, and the results are shown in Figure 3. The average heights of the ryegrass shoots in the control group were 16.4 cm, 23.8 cm, and 29.2 cm, and the heights of those with added NHAP were 16.6 cm, 24.4 cm, and 29.8 cm, respectively. The addition of Pb did not cause obvious toxicity to the growth of ryegrass; only a slight inhibition. The total fresh weight of ryegrass with NHAP showed a significant increasing trend compared with the control group, and there was a higher increase in root weight compared to shoot weight. The fresh weight of shoot increased by an average of 12.35%, while that of the root was 32.76%. Ryegrass has very large and dense fibrous roots which spread to the entire soil core in the pots during the experimental period. NHAP reduced the mobility and bioavailability of Pb, and alleviated the high toxicity of Pb to ryegrass. The P content in the soil was elevated after the addition of NHAP, which could promote plant growth and increase biomass. Thus, NHAP did not hinder the growth of ryegrass, but had a positive role in promoting its growth.

### 3.5. Organic Acid Response to Nano-Hydroxyapatitein the Ryegrass Rhizosphere

**Figure 4:**
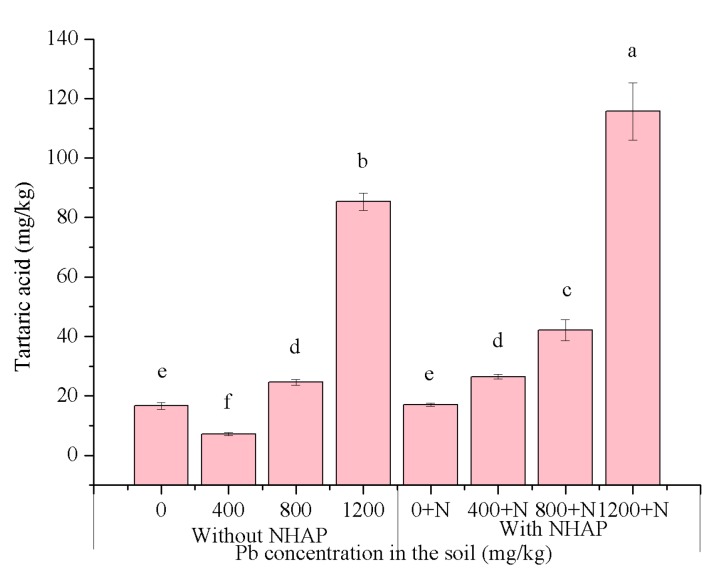
Tartaric acid contents in ryegrass rhizosphere soil under different treatments. a–f: The different letters in the table represent significant differences between treatments at *p* < 0.05.

Organic acids are widely present in plants and in the rhizosphere environment [30]. Under environmental stresses, such as heavy metals, organic acids secreted by plants were found to be significantly increased [31]. In this study, the low molecular weight organic acids in rhizosphere soil were measured after harvest at day 30. Tartaric acid was detected in perennial ryegrass rhizosphere soil, while other organic acids were below the limits of detection. As shown in Figure 4, tartaric acid content increased significantly along with the increase in Pb content in the soil. The tartaric acid contents were significantly positively correlated to the soil Pb contents in all treatments. This showed that secretion of tartaric acid by ryegrass was sensitive to Pb stress. The application of NHAP caused an increase in tartaric acid content by an average of 98.82% compared with the treatments without the addition of NHAP. The probable cause for this was that NHAP administration promoted the growth of ryegrass. It has been reported that plant root secretion of organic acids can improve the mobility and bioavailability of heavy metals in soil [32,33]. The increase of tartaric acid content also increased the likelihood that ryegrass absorbed Pb from rhizosphere soil. The objective of NHAP application was to lower the bioavailability of Pb, which seemed to be inconsistent with the role of tartaric acid. It has been reported that low molecular weight organic acids, including acetic acid, malic acid, citric acid, and oxalic acid, promoted the adsorption of Pb^2+^ on the surface of NHAP [34]. Therefore, the increase in tartaric acid could be considered as a beneficial aspect of NHAP for plant growth and reducing the bioavailabiliy of Pb. Tartaric acid had no inhibition effect on the remediation results of NHAP.

## 4. Conclusions

This study illustrated that NHAP could significantly reduce the mobility and bioavailability of Pb. The addition of NHAP effectively reduced the exchangeable and reducible fractions of Pb in soil, and transformed them into oxidizable and residual Pb, limiting its mobility and bioavailability. NHAP could play a very large role in controlling and mitigating the dangers of Pb pollution for organisms and the environment. The Pb contents of shoots and roots decreased and soil pH did not change significantly with the addition of NHAP; moreover, NHAP promoted the growth of ryegrass and the secretion of tartaric acid. This also indicated that the application of NHAP was beneficial to the growth of plants, and did not have negative impacts on the environment. The results in this study showed that NHAP could immobilize Pb in contaminated soil effectively, and can benefit the growth of ryegrass.

## Figures and Tables

**Table 1:** Design of the pot experiments for the different treatments.

Treatment	Pb-Spiked Content (mg/kg)	Addition Amount of NHAP (g)
0 mg/kg	0	0
0 mg/kg + NHAP	0	1.5
400 mg/kg	400	0
400 mg/kg + NHAP	400	1.5
800 mg/kg	800	0
800 mg/kg + NHAP	800	1.5
1200 mg/kg	1200	0
1200 mg/kg + NHAP	1200	1.5

NHAP: nano-hydroxyapatite.

**Table 2:** Sequential extraction procedure for soil Pb.

Fraction	Reagent	Shaking Time and Temperature
Exchangeable (F1)	40 mL of 0.11 mol/L CH_3_COOH	16 h at 25 °C
Reducible (iron/manganese oxyhydroxides) (F2)	40 mL of 0.5 mol/L NH_2_OH·HCl	16 h at 25 °C
Oxidizable (organic matter and sulfides) (F3)	10 mL of 8.8 mol/L H_2_O_2_, twice, cool and add 50 mL of 1 mol/L NH_4_Ac	1 h at 25 °C, 1 h at 85 °C, 1 h at 85 °C, 16 h at 25 °C
Residual (R)	HNO_3_-H_2_O_2_-HF	Microwave digestion

**Table 3:** Effects of NHAP on rhizosphere soil pH. The different letters in the table represent significant differences between treatments at *p* < 0.05.

Exogenous Pb Concentration (mg/kg)	The Rhizosphere Soil pH	
	Without NHAP	With NHAP
0	8.64 ± 0.10 ^a,b^	8.66 ± 0.03 ^a^
400	8.69 ± 0.04 ^a^	8.73 ± 0.02 ^a^
800	8.51 ± 0.20 ^b^	8.64 ± 0.03 ^a,b^
1200	8.12 ± 0.01 ^d^	8.20 ± 0.01 ^c^

^a^, ^b^, ^c^, ^d^: The different letters in the table represent significant differences between treatments at *p* < 0.05.
